# Effects of Blood-Flow Restriction During Body-Weight Semi-Squats on Post-Conditioning Drop-Jump Performance in Adolescent Female Volleyball Players

**DOI:** 10.3390/sports14070275

**Published:** 2026-07-02

**Authors:** Marcos Michaelides, Elena Mosfilioti, Gabriela Souza de Vasconcelos, Koulla Parpa, Konstantina Intziegianni, Evaggelos Nikolaou, Milto Hadjikyriakou, Marco Beato

**Affiliations:** 1 School of Sciences, University of Central Lancashire, Larnaca 7080, Cyprus; mmichaelides@uclan.ac.uk (M.M.); emosfi01@gmail.com (E.M.); kintziegianni@uclan.ac.uk (K.I.); nicolaou@rocketmail.com (E.N.); mhadjikyriakou@uclan.ac.uk (M.H.); 2 Department of Functional Health, Institute of Tropical Pathology and Public Health (IPTSP), Federal University of Goiás (UFG), R. 235, S/n, Goiânia 74605-050, Goiás, Brazil; vasconcelos.gs@gmail.com; 3 Department of Education and Sport Sciences, Pegaso Telematic University, Centro Direzionale Isola F2, 80143 Napoli, Italy; marcobeato.coach@gmail.com

**Keywords:** neuromuscular performance, warm-up, female volleyball, arterial occlusion pressure, body-weight semi-squats

## Abstract

This study investigated whether body-weight semi-squats (BWSSs) performed under blood-flow restriction (BFR) conditions were associated with higher post-conditioning drop-jump performance compared with the sham condition in adolescent female volleyball players. Thirteen players completed two experimental conditions (BFR and sham) in a randomized, counterbalanced crossover design. A pre-conditioning baseline jump assessment was not included. The BFR condition consisted of three sets of 16 repetitions with 80% of arterial occlusion pressure (AOP), whereas the sham condition was performed at 20% AOP. In each condition, participants performed one drop jump at 3, 6, 9, 12 and 15 min after the activation protocol. Jump height, impulse, reactive strength index (RSI) and power-related variables were analyzed using repeated-measures ANOVA. A significant main effect of condition indicated that jump height, RSI, impulse, and power-related variables were greater following the BFR than the sham condition across the post-conditioning assessment period. A significant main effect of time was also observed, with lower performance at 3 min compared to later time points. No condition-by-time interaction was found. These preliminary findings suggest that BWSSs performed under BFR conditions were associated with higher post-conditioning drop-jump performance than the sham condition. Low-load BFR exercise may therefore represent a practical strategy when heavy resistance equipment is unavailable. However, because no pre-conditioning baseline assessment was included, the magnitude of performance enhancement from baseline cannot be determined. Therefore, the findings should be interpreted as differences in post-conditioning performance between BFR and sham conditions rather than definite evidence of baseline to post enhancement.

## 1. Introduction

Previous research suggests that power-based movements may be enhanced when preceded by high-load exercise [1,2]. Heavy dynamic exercises, including ballistic and plyometric activities, have been used as conditioning to potentiate movements such as jumping, throwing, sprinting, changing direction, and kicking [3,4,5]. This acute improvement in strength and power performance following these conditioning activities has been described using multiple terms, with the most commonly used being post-activation performance enhancement (PAPE) [6].

The exact physiological mechanisms underlying this temporary enhancement in voluntary muscle performance remain unclear and are likely multifactorial. Although phosphorylation of myosin regulatory light chains has been proposed as one mechanism contributing to post-activation performance enhancement, this mechanism appears more closely related to electrically evoked twitch responses and may not fully explain voluntary performance enhancement in explosive tasks [7,8]. Similarly, neural mechanisms, including increased reflex sensitivity, α-motoneuron excitability, and voluntary neural drive, may contribute to the increased rate of force development and maximal force output following conditioning activities [9,10,11,12]. However, PAPE should not be attributed solely to phosphorylation-related or neural mechanisms. Recent evidence suggests that PAPE may also involve increased muscle temperature, enhanced blood flow, changes in muscle water content, and metabolic alterations, such as changes in pH [11,13]. Therefore, PAPE is best interpreted as an improvement in voluntary force or performance following a conditioning activity, arising from multiple interacting mechanisms rather than a single physiological pathway [14,15].

To introduce a post-conditioning performance response, as mentioned above, maximum or submaximal heavy conditioning activities can be used, but recently, blood-flow restriction (BFR) conditioning has been proposed as an alternative, which requires lower intensity to induce PAPE [16,17]. Several studies have suggested that low-load BFR exercise may increase recruitment of higher-threshold motor units as fatigue develops under reduced oxygen availability [18]. These findings help support the rationale that some neuromuscular responses during low-intensity BFR exercise may resemble those observed during traditional high-intensity resistance training, especially in relation to type II muscle fiber activation [18,19,20]. Dynamic activities incorporating BFR as part of the warm-up have been shown to be a promising method to potentiate the muscles involved in the subsequent activity [16]. Wang and colleagues demonstrated that BFR may improve subsequent lower-limb performance [21]. However, the optimal characteristics of BFR-based conditioning protocols for including PAPE remain unclear, including the exercise mode, cuff pressure, work volume, recovery duration, and outcome measures used to assess performance enhancement. The application of BFR-based conditioning strategies to volleyball performance remains limited, despite volleyball’s reliance on repeated high-intensity stretch–shortening cycle actions such as blocking and spiking [17,22]. Therefore, examining the acute effects of a BFR-enhanced PAPE stimulus on female volleyball athletes may help determine whether it offers meaningful performance advantages in this athletic population.

Thus, the primary purpose of the current study was to investigate whether BFR applied during body-weight semi-squats (BWSSs) would result in higher post-conditioning drop-jump performance compared with the sham condition. The secondary purpose was to examine the time course of post-conditioning jump performance during the 15 min following the conditioning protocol. It was hypothesized that BWSSs performed under BFR would be associated with higher post-conditioning drop-jump performance than the sham condition across the post-exercise assessment period.

## 2. Materials and Methods

This study used a randomized, counterbalanced, within-subject crossover design comparing BFR and sham conditions. The primary objective was to compare post-conditioning drop-jump performance between conditions across a series of randomized post-conditioning assessment time points. Because the study focused on post-conditioning responses, baseline jump measurements were not included prior to the conditioning protocol. Consequently, the design permits comparison of post-conditioning performance between conditions but does not permit quantification of within-condition changes from baseline.

### 2.1. Participants

Thirteen healthy adolescent female volleyball players voluntarily participated in this exploratory crossover study. All participants were competitive club-level athletes competing in official age-group volleyball competitions during the in-season period. They trained in a structured high-performance developmental environment, completing approximately four volleyball-specific training sessions and one official game per week. The average training background was approximately 5–6 years, and all had prior supervised resistance-training experience as part of their regular physical preparation.

To be eligible for inclusion, participants had to be female volleyball players aged 15–17 years, actively involved in regular volleyball training and competition, fully participating in team training at the time of testing, and familiar with supervised resistance training. All participants were free from musculoskeletal injury and had no known cardiovascular, neurological or other medical contraindications to blood-flow restriction exercise. The menstrual cycle phase was not monitored or controlled during the study. Athletes were excluded if they reported current pain or injury, any recent lower-limb injury that could affect jumping performance, recent surgery, absence from regular training or use of medication that could influence neuromuscular performance. The research was approved by the National Committee of Bioethics (EEBKEP 13 December 2024) and conducted in accordance with the Declaration of Helsinki. All participants were informed about the research background and the potential risks and benefits associated with the study. Written parental consent and participant assent were obtained before participation.

### 2.2. Procedures

Each participant completed one familiarization session and two randomized experimental sessions (BFR and sham) over a 21-day period. The familiarization session was conducted one week prior to the experimental sessions during which the participants practiced the drop-jump (DJ) technique from a 30 cm Stroops^®^ Ergo Plyo-box (Stroops, Inc., Clearfield, UT, USA). They were allowed to perform practice DJs until a consistent and technically correct execution was achieved. Anthropometric measurements were also obtained during this session. Stature was measured using a wall stadiometer (The Leicester^®^ Tanita^®^, Tokyo, Japan), and body mass was measured using a digital scale (C 418 MA, Tanita^®^, Tokyo, Japan).

The two experimental sessions consisted of the BFR and sham conditions and were conducted one week apart, consistent with previous BFR-based post-conditioning crossover research using a 7-day separation between conditions to control for biological variation [16]. The order of conditions was randomized and counterbalanced, with 6 participants completing BFR first and 7 participants completing the sham first. Sessions were scheduled at least 72 h after the athletes’ official game to account for fatigue and delayed-onset muscle soreness, and resistance training was avoided for 72 h before testing, as previously recommended [23]. All trials were conducted within similar daytime frames to reduce the potential influence of circadian rhythms. The order of the experimental conditions was randomized and counterbalanced using a computer-generated randomization sequence, with allocation performed by a researcher not involved in testing or outcome assessment. Due to the perceptible difference in cuff pressure between conditions, participants could not be fully blinded to the condition; however, both conditions used identical cuff placement and exercise procedures.

At the beginning of each experimental session, participants completed a standardized warm-up consisting of five minutes of running, followed by 5 min of dynamic stretching targeting the hamstrings, quadriceps and triceps surae muscles, and 10 submaximal vertical jumps (5 squat jumps and 5 countermovement jumps). After the warm-up, participants completed the assigned conditioning protocol. Following the conditioning activity, one DJ was performed at 3, 6, 9, 12, and 15 min post-activation, resulting in five DJs per condition. A pre-conditioning jump assessment was not included in the study design. Only one DJ was performed at each time point to minimize additional-testing related fatigue and potential interference with subsequent post-conditioning assessments.

**Figure 1:**
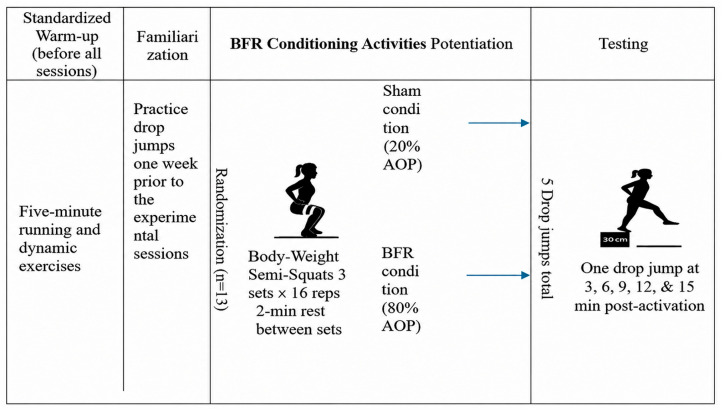
Experimental protocol flowchart: BFR: blood-flow restriction; PAPE: post-activation performance enhancement; AOP: arterial occlusion pressure.

During the DJs, participants were instructed to assume an upright stance with a straight torso, fully extend their knees, keep their hands on their hips, shoulder-width apart, and hold the position for at least 2 s before the drop phase. Jump performance was measured using a contact mat (SmartJump; Fusion Sport, Coopers Plains, Australia) to measure the jumping performance. Data with unwanted forward or sideways movements were removed from the analysis to avoid intentionally altering flight time and vertical jump height. Failure to land with both feet simultaneously on the jumping mat resulted in a failed attempt, and the trial was repeated. Participants were instructed to minimize ground contact time and jump as high as possible. Jump height was calculated from flight time using the SmartJump system. Reactive Strength Index (RSI) was calculated as jump height divided by ground contact time. Impulse and power output were obtained from SmartJump software version 1.0 (Smartspeed LITE software, Fusion Sport, Belgium). According to the Fusion Sport SmartJump user guide, impulse represents net upward impulse/net upward momentum derived from take-off velocity, whereas peak power output was calculated using the equations of Sayers et al. [24]. Relative power was calculated as peak power output divided by body mass. Because impulse and power were derived from contact-mat time-domain data rather than directly measured force-platform data, these variables were interpreted as secondary estimated outcomes. The same testing sequence was followed in both experimental sessions. Figure 1 summarizes the experimental design.

### 2.3. BFR Protocol

A pressure of 80% AOP was selected because BWSSs represent a low-load exercise stimulus, and higher relative pressures may be required to provide sufficient BFR stimulus during low-load dynamic exercise [25]. Following 3 min of rest, lower-limb AOP was measured individually to establish the target pressure for the BFR condition. For this purpose, the MAD-UP Pro^®^ (MAD-UP, Angers, France) cuffs were applied to the most proximal area of the lower limbs. The system has an automated, personalized pneumatic tourniquet system that maintains the pressure constantly despite the participant’s change in position. The target pressure was individualized to each participant’s physiological characteristics, as individualized occlusion pressure is considered important for both efficacy and safety. For the sham condition, the cuffs were applied in the same manner, but pressure was set at 20% AOP [26]. Although 20% of AOP was used as a sham/low pressure comparator to replicate cuff placement and participant experience, this pressure should not be considered completely inert. Therefore, the comparison in the present study is best interpreted as high-pressure BFR (80% AOP) versus low-pressure cuff application (20% AOP) rather than BFR versus no restriction.

### 2.4. Conditioning Protocol

In both conditions (BFR and sham), participants performed body-weight semi-squats to a depth corresponding to a 90-degree knee angle, using a 2:1 eccentric-to-concentric movement tempo [27]. Three sets of 16 repetitions were performed with a 2 min rest period between sets based on the protocol of Doma et al. [16], who used three sets of eight repetitions per leg, equivalent to 16 repetitions per set, during body-weight lower-limb exercise with BFR to induce post-activation performance enhancement and improve vertical jump performance. This volume was also considered appropriate for providing a sufficiently low-load conditioning stimulus while limiting excessive fatigue in adolescent female athletes. In both conditions (BFR and sham), cuff pressure was applied only during each set of the conditioning activity and released during the 2 min rest periods between sets. After the completion of the conditioning protocol, participants performed one DJ at 3, 6, 9, 12 and 15 min post-activation.

### 2.5. Statistical Analyses

The design of this study was a within-subjects crossover protocol with repeated measures. The data were analyzed using IBM^®^ SPSS^®^ Statistics 28 (IBM Corporation, Chicago, IL, USA). The data normality was assessed using the Shapiro–Wilk test. Data were presented as mean ± standard deviation (SD). Data were analyzed using a 2 × 5 repeated-measures ANOVA, with condition (BFR vs. sham) and time (3, 6, 9, 12 and 15 min) as within-subject factors to explore differences between the BFR and the sham conditions in jump height, RSI, impulse, absolute and relative power. Accordingly, statistical analyses were designed to evaluate differences in sport-conditioning performance between conditions and across time points rather than changes from a pre-conditioning baseline. When the assumption of sphericity was violated, Greenhouse–Geisser corrections were applied. F-values and ηp^2^ effect sizes were reported for the overall ANOVA analysis and interpreted as follows: small ηp^2^ ≈ 0.01, medium ηp^2^ ≈ 0.06, and large ηp^2^ ≈ 0.14 [28]. To facilitate comparison with previous BFR-PAPE literature, standardized mean differences were also calculated using Hedges’ gav. Hedges’ gav was calculated as the between-condition mean difference divided by the average standard deviation of the two conditions, with a small-sample correction applied. Standard deviations were derived from the reported standard errors using SD = SE × √n. Bonferroni-adjusted pairwise comparisons were used for post hoc analyses. Significance was set at *p* < 0.05. Because no a priori sample size calculation was performed, a post hoc analysis was conducted for the primary outcome, jump height. Based on the observed condition effect for jump height (ηp^2^ = 0.55), the achieved statistical power was 0.99. This analysis was conducted to provide context regarding statistical sensitivity; however, post hoc estimates should not be considered a substitute for prospective sample size estimation and should be interpreted with caution. Intra-session reliability for repeated drop-jump height measures was assessed across five post-conditioning jumps using a two-way mixed-effects, average measures interclass correlation coefficient [ICC(3,k)]. Drop-jump height demonstrated excellent reliability in both conditions, with ICC(3,k) = 0.926, CI 95% = 0.814–0.975 for the BFR condition and ICC(3,k) = 0.905, 95% CI = 0.788–0.967 for the sham condition. These values indicate that the SmartJump contact mat provided reliable repeated drop-jump height measurements in the present sample.

## 3. Results

Thirteen healthy adolescent female volleyball players participated in the study. Their anthropometric characteristics are presented in Table 1.

The repeated-measures ANOVA demonstrated that there was no significant condition-by-time interaction for the reported variables (jump height, impulse, RSI, absolute power, and relative power), indicating a similar pattern of change over time between the BFR and sham conditions. Therefore, the main effects of condition and time were examined.

### 3.1. Jump Height

**Figure 2:**
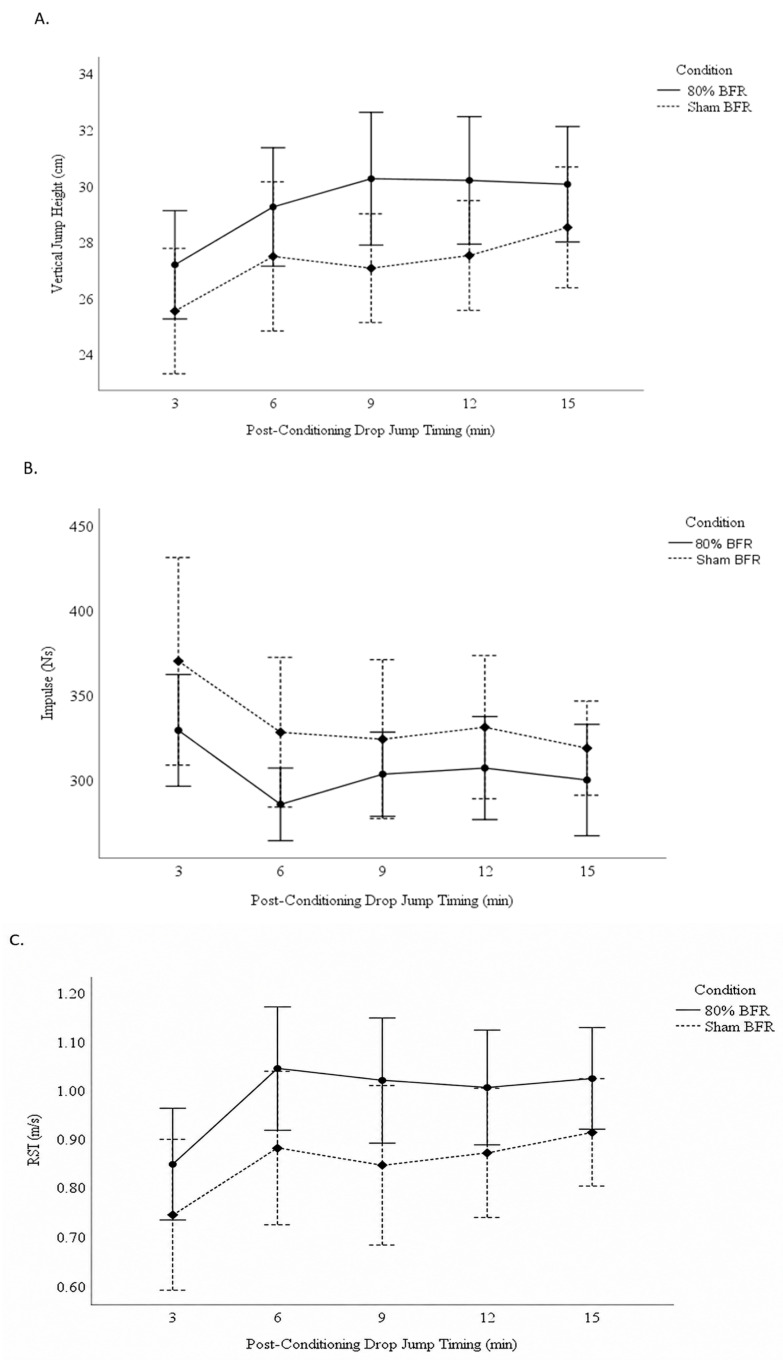

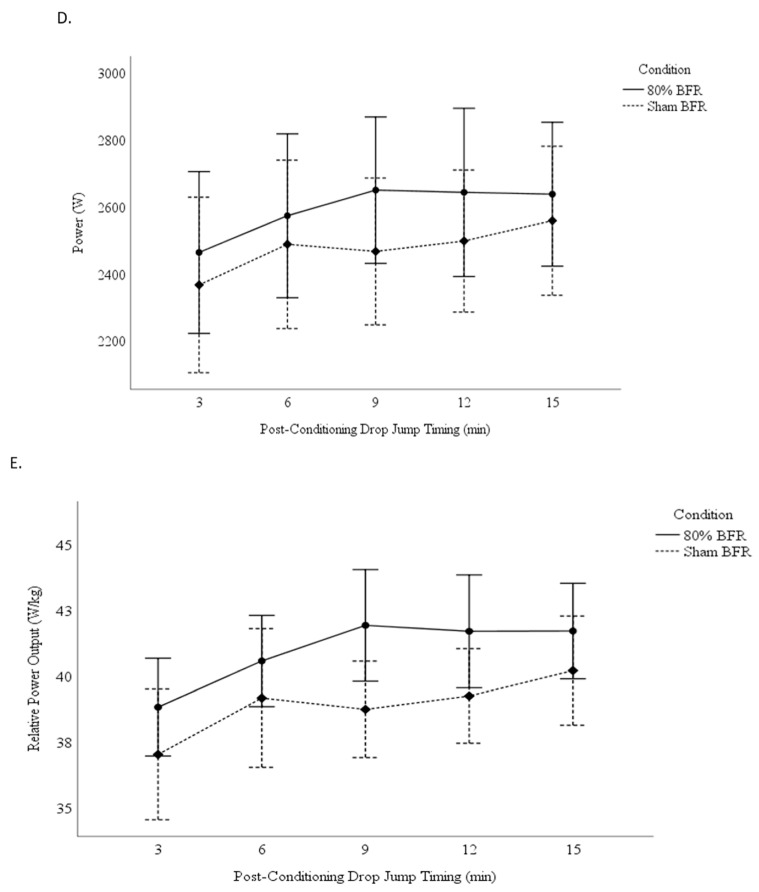
(**A**–**E**) Mean values of BFR and sham conditions for jump height (**A**), impulse (**B**), reactive strength index (**C**), absolute power (**D**), and relative power (**E**) across the five post-activation time points (3, 6, 9, 12, and 15 min). BFR = blood-flow restriction; error bars represent 95% CIs.

A significant main effect of condition was observed for jump height [F(1,12) = 14.56, *p* < 0.01, ηp^2^ = 0.55], indicating that jump height was significantly greater following the BFR condition (M = 29.39 ± 0.90 cm, 95% CI = 27.41–31.36 cm) compared to the sham condition (M = 25.30 ± 0.88 cm, 95% CI = 23.38–27.22 cm). A significant main effect of time was also observed [F(4,48) = 7.79, *p* < 0.01, ηp^2^ = 0.39]. Post hoc analysis revealed that jump height at 3 min was significantly lower than at the subsequent post-conditioning time points, after which values remained relatively stable. The standardized between-condition effect for jump height was large (Hedges’ gav = 1.19). The absence of a significant interaction indicates that the temporal pattern was similar in both conditions, although jump height remained higher in the BFR condition across measurements (see Figure 2A).

### 3.2. Impulse

A significant main effect of condition was observed for impulse [F(1,12) = 5.98, *p* < 0.05, ηp^2^ = 0.33], indicating that it was significantly greater following the BFR condition (M = 151.29 ± 5.32 Ns, 95% CI = 139.70–162.69 Ns) compared to the sham condition (M = 146.62 ± 5.0 Ns, 95% CI = 135.73–157.50 Ns). Additionally, a significant main effect of time was observed [F(4,48) = 7.42, *p* < 0.01, ηp^2^ = 0.36] with no significant interaction. The standardized between-condition effect for impulse was small (Hedges’ gav = 0.24). Post hoc comparisons indicated that impulse at 3 min was significantly lower at the subsequent post-conditioning time points, with no further differences observed thereafter (see Figure 2B).

### 3.3. Reactive Strength Index (RSI)

No significant condition-by-time interaction was identified for RSI. A significant main effect of condition was observed [F(1,12) = 11.86, *p* < 0.01, ηp^2^ = 0.50], with RSI significantly greater following the BFR condition (M = 0.99 ± 0.05 m/s, 95% CI = 0.88–1.1) compared to the sham condition (M = 0.85 ± 0.06 m/s, 95% CI = 0.73–0.98). A significant main effect of time was also observed [F(4,48) = 10.75, *p* < 0.01, ηp^2^ = 0.47]. The standardized between-condition effect for RSI was moderate (Hedges’ gav = 0.66). Post hoc comparisons revealed that RSI at 3 min was significantly lower than at 6, 9, 12, and 15 min, with no further differences observed between later post-conditioning assessments (see Figure 2C).

### 3.4. Absolute Power

No significant condition-by-time interaction was observed for absolute power, indicating a similar temporal pattern of change between the BFR and sham conditions. However, there was a significant main effect of condition [F(1,12) = 7.51, *p* < 0.05, ηp^2^ = 0.40]. Across time, absolute power was significantly greater following the BFR condition (M = 2592 ± 105.00 W, 95% CI = 2364.08–2821.63 W) compared to the sham condition (M = 2474.79 ± 103.06 W, 95% CI = 2250.24–2699.00 W). A significant effect of time was identified [F(1.66,19.89) = 7.54, *p* < 0.05, ηp^2^ = 0.36]. The standardized between-condition effect for absolute power was small (Hedges’ gav = 0.29). Post hoc analyses indicated that absolute power at 3 min was significantly lower than at the later post-activation time points, indicating that absolute power was lower at 3 min than at subsequent post-conditioning assessments with no further differences observed thereafter (see Figure 2D).

### 3.5. Relative Power

No significant condition-by-time interaction was observed for relative power, indicating a similar temporal pattern of change between the BFR and sham conditions. A significant main effect of condition was observed [F(1,12) = 12.76, *p* < 0.01, ηp^2^ = 0.60], demonstrating significantly greater relative power following the BFR condition compared to the sham condition. The estimated marginal means showed consistently greater relative power values across all post-activation jumps with BFR (M = 40.94 ± 0.80 W/kg, 95% CI = 35.90–42.80 W/kg) compared to the sham condition (M = 38.87 ± 0.87 W/kg, 95% CI = 34.50–42.30 W/kg). Additionally, a significant effect of time was identified [F(1.69,20.28) = 7.49, *p* < 0.05, ηp^2^ = 0.38]. The standardized between-condition effect for relative power was moderate (Hedges’ gav = 0.64). Post hoc analyses indicated that relative power at 3 min was significantly lower than at the subsequent post-activation time points, with no further differences observed thereafter, indicating that relative power was lower at 3 min than at the subsequent post-conditioning assessments, with no further differences observed thereafter (see Figure 2E).

## 4. Discussion

The purpose of the study was to examine whether post-conditioning drop-jump performance differed between BFR and sham conditions following a standardized body-weight semi-squat protocol. The main finding was that jump height, RSI, impulse, and power-related variables were consistently greater following the BFR condition than following the sham condition. No condition-by-time interactions were observed, indicating that the temporal pattern of responses across the post-conditioning assessment period was similar between conditions. Therefore, the findings are best interpreted as an overall condition effect, whereby performance outcomes were higher following BFR than sham across the post-conditioning assessment period. Because no pre-conditioning baseline assessment was included, the present findings do not permit quantification of baseline-to-post changes and should not be interpreted as direct evidence of the magnitude of PAPE within either condition. Furthermore, the observed differences cannot determine whether BFR enhanced performance above baseline or whether both mechanisms contributed to the findings.

These findings differ from previous studies [29] reporting similar effects at lower AOPs, suggesting that pressures greater than 70% AOP may not provide additional benefits and may induce earlier fatigue. However, the present findings indicate that 80% AOP was associated with consistently greater performance compared to the sham condition across the 15 min post-exercise period (see Figure 2A–E). These findings are in agreement with Doma et al. [16], who applied an occlusion pressure of 130% of systolic blood pressure. Although their protocol differed (unilateral exercises), they similarly reported improvements in jump height and absolute power within the 6–15th min post-exercise. In the current study, performance was consistently higher overall in the BFR condition across the assessment period. Although the observed condition effect sizes were larger than those reported in some previous BFR-PAPE studies [17,29], these values should be interpreted within the context of the repeated-measures crossover design used in the present study, which reduced between-subject variability and may increase the partial eta squared estimated. Furthermore, the absence of a significant condition-by-time interaction indicates that the findings reflect overall differences between conditions rather than distinct temporal responses.

While previous investigations have reported improvements in jump performance following BFR-based conditioning, the present study demonstrated consistently higher post-conditioning values for several performance variables, including RSI and impulse, in the BFR condition compared with the sham condition. RSI is calculated as jump height divided by ground contact time; therefore, higher RSI values reflect the ability to achieve greater jump height while minimizing contact time. This is commonly interpreted as an indicator of stretch–shortening cycle efficiency, which is essential for rapid transitions from eccentric to concentric muscle actions [30]. These findings are highly relevant to volleyball, as performance depends on repeated stretch–shortening cycle actions, including jumping, blocking, and spiking.

A recent study on male volleyball players by Wei et al. [17] aligns with our findings, as they also demonstrated that low resistance exercises combined with BFR elicited greater PAPE than the same exercises performed under high resistance. Further to that, they also demonstrated that potentiation occurred 4–20 min post-exercise, but with peak benefits between 8 and 16 min. The long-lasting effects of BFR on PAPE are of high importance, especially in female volleyball matches, which demonstrated longer rally durations compared to those observed in men [31]. Enhanced impulse may also have practical relevance in volleyball, as it may contribute to more efficient eccentric and concentric phases during repeated jumping actions [22,32].

Two key factors must be considered when attempting to maximize post-activation performance: the similarity between the conditioning activity and the subsequent task, and the balance between fatigue and potentiation [28,33]. These two factors may be influenced by the intensity of conditioning activities executed [34], the length of rest periods and the individuals’ experience [35]. For instance, Crewther et al. [36] demonstrated that heavy back squats (3-reps at 90–95% of 1RM) potentiated explosive activities such as sprinting and jumping. These effects were apparent four min post-recovery, indicating the importance of the recovery timing. These results varied based on athleticism since stronger athletes benefited more from the PAPE protocol [36]. Whether similar or larger between-condition differences would be observed in stronger, older, or male athletes remains unknown and requires further investigation.

In the present study, performance at the first measurement point (3 min post-conditioning) was lower than at the subsequent time points in both conditions. This finding suggests that the earliest post-conditioning assessment may have occurred before participants had fully recovered from the conditioning protocol. Similar temporal patterns have been reported in previous studies examining conditioning activities and subsequent jump performance [16,36]. Although interaction between fatigue and post-activation-related processes has been proposed as a potential explanation for such responses, the physiological mechanisms underlying the present findings were not directly assessed, and therefore any mechanistic interpretation should be considered speculative. The interaction between fatigue and mechanisms proposed to underlie post-conditioning performance responses has been discussed extensively in the previous literature [37,38]. Evidence suggests that fatigue dissipates over time, allowing performance differences to become more evident [16,39,40]. Therefore, the improved performance observed at later time points may reflect the combined influence of reduced residual fatigue and other PAPE-related mechanisms, rather than a single underlying mechanism.

From a practical perspective, the observed improvements in jump height, RSI, impulse, and power-related variables may be meaningful for volleyball performance because these qualities contribute to explosive actions such as spiking, blocking, and rapid changes in movement direction. The findings also suggest that low-load BFR exercise may represent a practical conditioning strategy when access to heavy resistance equipment is limited or when minimizing mechanical loading is desirable. Importantly, the present findings should be interpreted as evidence of differences in post-conditioning performance between experimental conditions rather than evidence of baseline-to-post performance enhancement.

Several limitations warranted consideration in the current study. The relatively small sample size (n = 13) and the inclusion of a specific population (adolescent female volleyball players) may limit the generalizability of the presented findings to other athletic populations. Also, multiple related performance outcomes were analyzed using separate repeated-measures ANOVAs. Although these variables represented closely related indices derived from the same drop-jump task, the use of multiple statistical tests may have increased the risk of type I error. In addition, no a priori power analysis was performed, and the post hoc power analysis should be interpreted cautiously because it does not replace prospective sample size estimation. Another major limitation was the absence of a traditional high-load conditioning comparison, which is commonly used to elicit PAPE. Furthermore, the absence of monitoring the athletes’ menstrual cycles may have introduced additional variability in neuromuscular performance and potentially influenced the observed responses.

Although the randomized, counterbalanced crossover design reduced the influence of stable between-participant differences, it did not eliminate potential day-to-day variation in physiological or psychological state. Factors such as sleep quality, nutrition status, residual fatigue, motivation and arousal were not directly monitored and may have influenced performance. In addition, because the difference in cuff pressure between conditions was perceptible, participants could not be fully blinded to condition allocation. Therefore, expectancy effects of placebo-related influences cannot be completely excluded, and the observed differences between conditions should not be attributed exclusively to the physiological effects of BFR. Furthermore, because the sham condition involved cuff inflation to 20% AOP, some degree of vascular restriction cannot be excluded. Thus, the present findings should be interpreted as a comparison between high-pressure BFR and low-pressure cuff application, rather than BFR versus a true no-restriction control.

A major limitation of the present study was the absence of a pre-conditioning performance assessment. Consequently, the study does not permit quantification of baseline-to-post changes in performance and does not satisfy recent methodological recommendations for PAPE research [41]. Future studies should include appropriate pre-conditioning assessments and, where possible, assessments before and after warm-up to distinguish warm-up-related effects from conditioning-related effects.

A strength of the present study is the use of a controlled and practically applicable protocol. Despite the small sample size, the observed differences in power-related variables are meaningful considering the importance of lower-body power in volleyball. These findings support further investigation comparing BFR with traditional high-load conditioning strategies.

## 5. Conclusions

Body-weight semi-squats performed under BFR at 80% AOP were associated with higher post-conditioning jump performance than the sham condition in adolescent female volleyball players. Specifically, the BFR condition was associated with greater jump height, RSI, impulse and power-related outcomes across the post-conditioning assessment period.

These findings suggest that low-load BFR conditioning may have potential as a practical warm-up strategy in this population, especially when access to heavy resistance equipment is limited. However, because the present study did not include a direct comparison with a traditional high-load comparison condition, further research is required before BFR-SS can be recommended as an alternative to high-load conditioning strategies. Future research should investigate the effectiveness of this protocol in different populations and compare it directly with traditional high-load conditioning strategies.

## Figures and Tables

**Table 1:** Descriptive statistics for age, height, and weight.

	N	Mean ± SD
Age (years)		16.15 ± 0.80
Height (m)	13	1.68 ± 0.05
Weight (kg)		63.31 ± 8.03

## Data Availability

Data can become available upon request to the corresponding author.
